# The Dartmouth Database of Children’s Faces: Acquisition and Validation of a New Face Stimulus Set

**DOI:** 10.1371/journal.pone.0079131

**Published:** 2013-11-14

**Authors:** Kirsten A. Dalrymple, Jesse Gomez, Brad Duchaine

**Affiliations:** 1 Department of Psychological and Brain Sciences, Dartmouth College, Hanover, New Hampshire, United States of America; 2 Institute of Cognitive Neuroscience, University College London, London, United Kingdom; University of Udine, Italy

## Abstract

Facial identity and expression play critical roles in our social lives. Faces are therefore frequently used as stimuli in a variety of areas of scientific research. Although several extensive and well-controlled databases of adult faces exist, few databases include children’s faces. Here we present the Dartmouth Database of Children’s Faces, a set of photographs of 40 male and 40 female Caucasian children between 6 and 16 years-of-age. Models posed eight facial expressions and were photographed from five camera angles under two lighting conditions. Models wore black hats and black gowns to minimize extra-facial variables. To validate the images, independent raters identified facial expressions, rated their intensity, and provided an age estimate for each model. The Dartmouth Database of Children’s Faces is freely available for research purposes and can be downloaded by contacting the corresponding author by email.

## Introduction

Faces are important social stimuli and therefore a frequent focus of scientific investigation. They are used as stimuli in a variety of research areas, including emotion, social attention, speech perception, human face recognition, computer face recognition, eyewitness identification, and in the study of neuropsychological disorders such as autism and prosopagnosia. Within the field of face recognition alone, research covers a breadth of topics including expression recognition, identity perception and memory, gender discrimination, age recognition, and uses methods that range from behavioral testing to neuroimaging and neuropsychology. Access to a well-controlled set of face stimuli is critical to experimental design, and effects such as the own-age bias [1]–[3], where individuals are better at remembering faces from their own age group, demonstrate that the age of faces can be an important consideration in stimulus selection.

Although several databases of adult face stimuli exist (see [4], for an excellent review), very few databases of children’s faces are available [5]. Of these, the most extensive is the NIMH Child Emotional Faces Picture Set (NIMH-ChEFS) [5], which includes front-facing images of sixty children between 10 and 17 years-of-age, posing five facial expressions with direct and averted gaze. This database provides a good variety of images, including faces of children of different races, multiple facial expressions and gaze directions, and visible extra-facial features such as hair, jewelry, and clothing. While this variety increases the usefulness of the database, it compromises certain aspects of stimulus control that may be desirable in some areas of study. Thus, there is a need for a freely available database of children’s faces that, while providing stimulus variety, places particular emphasis on stimulus control.

We aimed to produce such a database that will be useful for a broad spectrum of research applications. Our database includes images from multiple facial angles, expressions, and identities, from both male and female models between the ages of 6 and 16. We enhanced stimulus control by minimizing the possibility that individual faces can be identified based on extra-facial cues (e.g. hair, glasses, etc…) or characteristics of the images themselves (e.g. background, lighting, image quality, etc…). In other words, we aimed to make the images relatively homogenous with the exception of desired variations such as facial identity and expression.

To validate the images in our database, we asked independent raters to identify the facial expressions, rate their intensity, and estimate the age of each model. In this report we provide a detailed description of this freely available database, including the procedure for image acquisition and processing, and analysis and results of the validation procedure. We acknowledge that no database will fit the needs of all researchers, but our primary goal is to make the Dartmouth Database of Children’s Faces available in the hope that it will be useful across a range of research domains. Access to the database can be requested by emailing the corresponding author. (Note: researchers must sign a license agreement, agreeing to terms of use. Images are 900×900 pixels (300 dpi) in.jpg format).

## Methods

### 1. Development of Database

#### 1.1 Image acquisition

**Figure 1:**
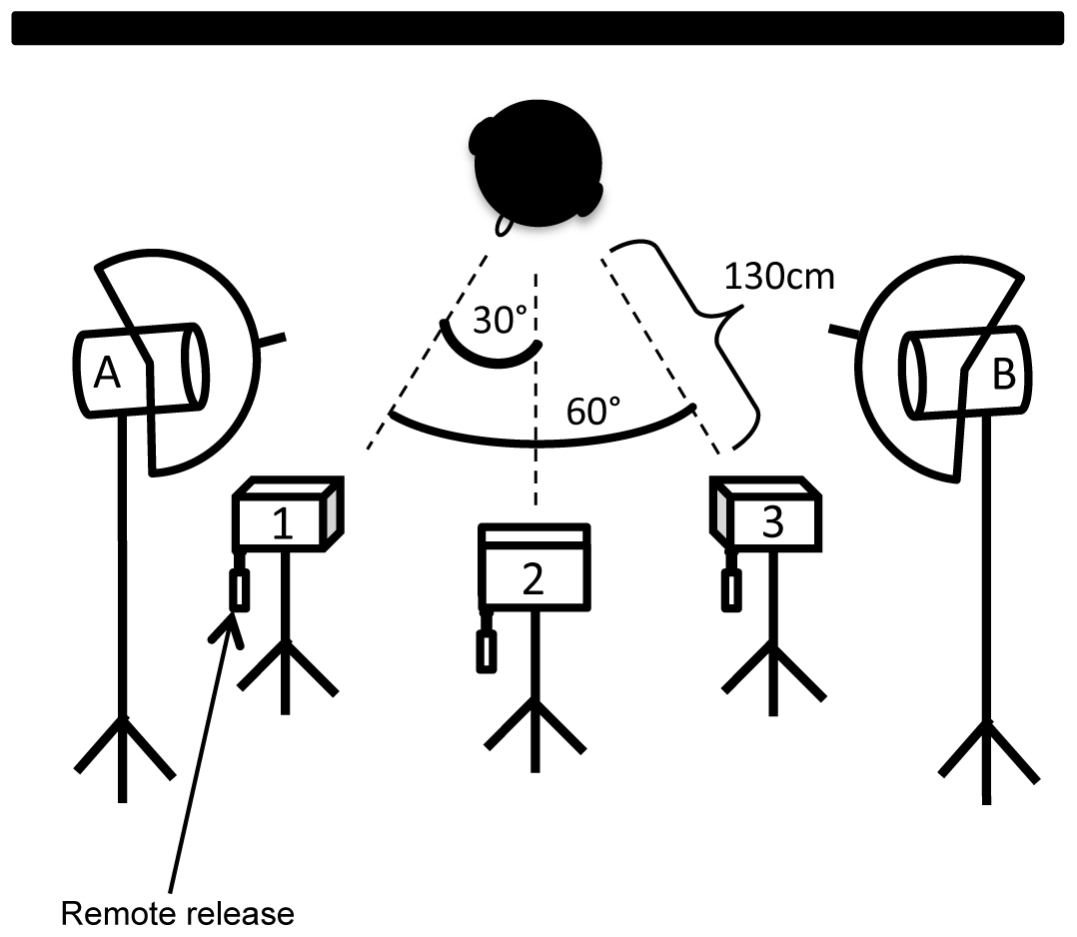
Camera set up. Models wore black hats to cover hair and ears and were seated in front of a black felt backdrop. Three cameras were positioned at 0°, 30° and 60°, and controlled using dot-line remote release triggers. Spotlights were positioned at 0° and 60° and were softened using white photography umbrellas. Models initially faced Camera 1. Once photographs were taken for all facial expressions, under two lighting conditions, models were reseated to face Camera 3, allowing 0°, 30° and 60°, angles of the other side of the face.

Photographs were taken at Dartmouth College and at the University of Minnesota using the same equipment and set up (Figure 1). Children between the ages of 5 and 16 (mean age 9.72, SD = 2.41) were recruited from the Dartmouth community and through the University of Minnesota database of child participants. Children (n = 123, 61 male) and their parents provided written assent and permission to have photographs taken and distributed to other researchers. They also specified whether photographs can be used in scientific publications and/or presentations (e.g. used in figures). Children were paid for their participation. Participation was in accordance with the ethical guidelines of the Committee for the Protection of Human Subjects at Dartmouth College.

Children were dressed in black salon gowns and black hats that covered their ears before being seated in front of a black felt backdrop. Glasses and jewelry such as necklaces and earrings were removed. Ceiling lights were kept on, but no camera flash was used. Instead, two spotlights with 250-Watt light bulbs lit the models from 30 degrees on each side of the central camera (0 and 60 degrees with respect to the model). Spotlights were softened with white photography umbrellas. Three Canon EOS Rebel XS cameras were positioned at a distance of 130 cm in front of the model at 0 degrees, and 30 and 60 degrees to the model’s left or right. Dot-line remote releases allowed the three cameras to be triggered simultaneously.

Each model was asked to pose eight different facial expressions: neutral, content (smile without teeth), happy (smile with teeth), sad, angry, afraid, surprised, and disgusted. Two happy expressions, which we call “happy” and “content”, were included to provide the option of having teeth visible or hidden (teeth can provide feature-based identity information) and to have a choice of intensity of the expression (happy with a large smile versus more subtle, “content”/“pleased”). Models were coached by encouraging them to imagine situations that would elicit the desired facial expressions (e.g. Disgust: “Imagine sitting in chewing gum”, or, Anger: “Imagine your brother or sister broke your PlayStation”), and photos were taken until the photographer felt satisfied that the expressions were the best the child could produce. Each facial expression was photographed at least twice and in two lighting conditions. One lighting condition used both spotlights, while the other used only one (Spotlight A, see Figure 1).

**Figure 2:**
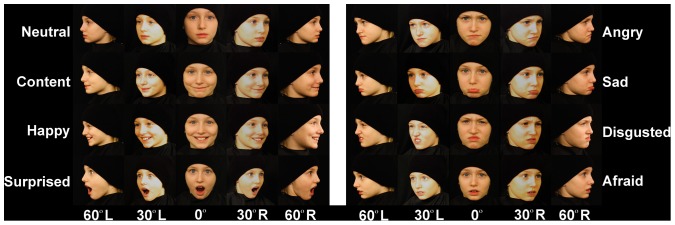
Sample photographs. Female model posing all eight facial expressions from five camera angles. The parent of this model provided written informed consent, as outlined in the PLOS consent form, for publication of their child’s photographs.

Models initially faced Camera 1 (see Figure 1). Once photographs were taken for all eight facial expressions, under two lighting conditions, the model was positioned to face Camera 3. The model was asked to produce all expressions again. Combined, this allowed for frontal, as well as 30 degree, and 60 degree angles of the each side of the model’s face. An example of all facial expressions and camera angles for one model are in Figure 2.

#### 1.2 Image processing

All images of all models were visually inspected for quality. Poor quality images (in which the model was moving, blinking, making an unidentifiable facial expression, or was out of focus) were removed. Due to the racial homogeneity in the Dartmouth community, the vast majority (118/123) of the children who volunteered to participate were Caucasian. Given the small number of other-race faces in our sample, we chose to restrict the image set to Caucasian models only, leaving images of 50 male and 51 female models. Images were cropped to 300×300 pixels (100 dpi) around the face and were otherwise left unprocessed.

### 2. Validation of the Database

#### 2.1 Participants

Students from Dartmouth College and members of the Dartmouth college community (n = 163, 96 female, mean age 19.6, SD = 4.15) rated the faces. Participants gave written consent and were compensated financially or with course credit. Participation was in accordance with the ethical guidelines of the Committee for the Protection of Human Subjects at Dartmouth College.

#### 2.2 Procedure

Participants rated at least one block of images, but no more than two blocks in one sitting. Blocks included all frontal images from 10 models (one block had 11 female models). Participants were seated at a comfortable viewing distance from a 13″ Macbook Pro laptop computer. For each block of models, they were asked to perform three tasks: identify the facial expression, rate the intensity of the facial expression, and estimate the model’s age.

Participants were first asked to identify the facial expression. The image appeared on the screen above seven possible expression words: neutral, happy, sad, angry, afraid, surprise, disgust. These words were displayed with a number corresponding to the key participants were to press to select that expression. The expression words were presented in a different order for each block of models. An additional option, “none” was included at the end of the list of expression words. Participants were instructed to choose “none” if they felt that the facial expression did not match any of the expression words. The experimenter explicitly stated that the word “neutral” represented a lack of facial expression, whereas “none” indicated that the facial expression was ambiguous. Content was not one of the choice expression words, so the correct classification for content facial expressions (happy with no teeth visible) was “happy”.

After images were classified based on facial expression, participants rated the same images for the intensity of the expression. Images were presented randomly and participants were asked to rate them on a scale of 1–5 with 1 representing low intensity, and 5 representing high intensity. Participants indicated their rating by key press. Finally, participants were asked to estimate the age of each model. They were first shown three example faces, a 5-year-old, a 10-year-old, and a 15-year-old, to familiarize them with what children of those ages look like. Models for these examples were chosen from other blocks of models. Age estimates were given for one neutral expression image per model and were limited to whole values in years (e.g. 9-years-old). Participants indicated their age estimate by typing it in an answer box on the screen and confirming their answer by pressing enter. Participants were given unlimited time for all ratings. The total time to perform all three tasks for one block of images was between 18–25 minutes.

## Analysis and Results

### 1. Expression Ratings

First, a score reflecting the identifiability of facial expressions was computed for each model. This score was based on the mean number of times the model’s posed facial expressions were correctly identified by the raters. These scores ranged from 54.7% to 90.6% meaning that the best model produced facial expressions correctly identified by 90.6% of the raters on average. Based on these scores, the bottom 10 male and 11 female models were removed from the image set, leaving the best 40 male and 40 female models. We chose to retain 40 male and 40 female models in order to maximize the number of identities in the database, while removing the models with the lowest ratings. The identifiability scores for the final set of models ranged from 70.0% to 90.6% (Table 1). The remaining analyses were performed using only these 80 models.

**Table 1:** True age, estimated age, and identifiability scores for top 40 male and top 40 female models.

Males					Females				
Rank	Model ID	True age (years)	Est. age (years)	Score (%)	Rank	Model ID	True age (years)	Est. age (years)	Score (%)
1	104	12	9.6	88.7	1	112	9	7.7	90.6
2	87	9	8.4	88.1	2	115	12	9.3	89.7
3	72	10	10.7	86.5	3	114	9	11.4	88.4
4	29	15	14.7	86.3	4	123	16	14.2	88.4
5	79	7	5.8	85.9	5	122	16	12.7	87.8
6	37	8	8.3	85.3	6	119	14	12.2	87.8
7	60	7	6.1	85.0	7	110	13	12.4	85.3
8	89	9	9.0	84.7	8	77	10	8.7	85.0
9	38	10	10.7	84.4	9	120	9	9.1	84.4
10	108	10	11.7	83.0	10	105	9	8.4	83.4
11	51	11	12.0	82.8	11	96	12	9.2	83.0
12	76	9	6.6	82.5	12	109	14	12.5	82.5
13	40	12	9.5	81.3	13	117	9	7.4	80.9
14	68	9	10.0	80.9	14	64	10	7.0	80.6
15	107	14	14.1	80.4	15	98	9	8.6	80.3
16	4	13	10.2	80.3	16	33	6	5.3	80.0
17	36	11	12.4	80.3	17	32	6	6.6	79.7
18	59	8	7.1	80.3	18	62	10	8.1	79.7
19	70	8	9.7	80.3	19	52	9	7.2	79.4
20	80	11	11.0	80.3	20	65	8	7.4	78.8
21	84	11	12.6	80.0	21	113	15	11.7	78.8
22	86	7	7.9	80.0	22	99	7	6.2	77.8
23	103	12	9.2	79.8	23	69	7	6.3	77.8
24	75	10	8.4	79.7	24	13	11	9.4	77.5
25	81	8	5.8	79.7	25	45	7	8.5	75.9
26	92	8	6.8	79.2	26	88	12	12.2	75.6
27	56	8	9.4	77.8	27	57	9	9.8	75.3
28	27	9	9.2	77.2	28	78	9	9.8	75.3
29	49	8	8.2	76.9	29	61	8	6.5	74.7
30	53	8	11.2	76.3	30	94	7	6.4	74.4
31	41	8	6.8	75.3	31	74	8	9.3	74.1
32	63	10	7.9	75.3	32	26	7	7.1	73.8
33	73	8	10.6	75.3	33	71	9	10.7	73.8
34	44	11	8.8	74.1	34	55	10	10.9	73.1
35	58	8	8.3	74.1	35	102	9	7.4	72.7
36	28	11	11.9	73.2	36	121	7	6.5	72.5
37	10	9	7.6	73.1	37	16	11	8.5	72.2
38	48	11	8.3	72.5	38	111	13	13.5	71.9
39	11	10	8.6	70.3	39	22	9	7.2	71.3
40	66	8	8.0	70.0	40	25	13	13.1	70.3
**Mean (SD)**		9.7 (1.9)	9.3 (2.1)	79.7 (4.8)	**Mean (SD)**		10.0 (2.7)	9.1 (2.4)	79.1 (5.7)

Age estimates are based on mean estimate from raters. Identifiability scores (%) are based on the mean number of times that the model’s posed facial expressions were correctly identified by the raters.

**Figure 3:**
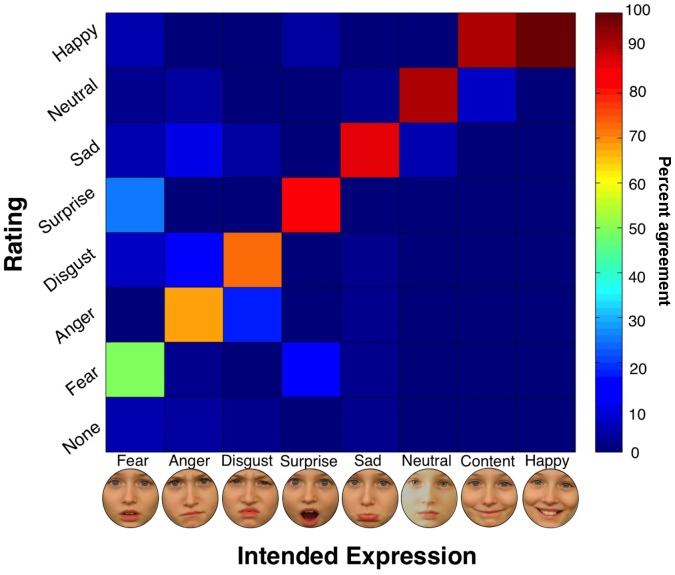
Confusability matrix. Rows: rater-chosen expressions. Columns: intended expressions. Diagonal represents agreement between rater-chosen expressions and intended expressions, with warmer colors representing greater agreement (red = 100% agreement, blue = 0% agreement). Off-diagonal cells represent confusability of intended expression for an alternate expression, with warmer colors indicating greater confusability. Expressions identified as “none” could not be classified (i.e. they were ambiguous). The parent of this model provided written informed consent, as outlined in the PLOS consent form, for publication of their child’s photographs.

A confusability matrix was computed, indicating the percent of accurate identifications of a given facial expression, and, if inaccurate, which expression was chosen (Figure 3). Cohen’s Kappa [6] indicated good agreement between rater-chosen expressions and intended expressions, Kappa = 0.780, 95% CI (0.775, 0.786). On average, the expressions were correctly identified in 79.7% of the images (SD = 22.7%), which is comparable to rates from other published face databases [7]. Happy and Content were the most accurately identified expressions; raters correctly classified 97.8% of the Happy (teeth visible) images and 90.8% of the Content (teeth not visible) faces. The least accurately identified expression was Afraid, which was correctly identified in 49.0% of the images, and most often confused with Surprised (26.0% of the Afraid images). This pattern is similar to that seen for ratings of Ekman and Friesen’s Pictures of Facial Affect [8], [9]. The mean rating for each model is indicated Table 1. Ratings for individual images can be found in the supplementary information (Table S1).

Others have found that the gender of the observer may affect memory for faces [10], [11] and other stimuli [12] and that these effects may interact with the gender of the face stimuli [13]. We therefore performed a 2×2 analysis of variance (ANOVA) on the accuracy ratings with factors of Rater (Male vs. Female) and Model (Male vs. Female). There was a main effect of Rater gender such that Female raters were more accurate at identifying the intended expression (mean = 80.6%, SD = 23.5%) than Male raters (mean = 78.3%, SD = 25.5%), F(1,2567) = 5.66, *p* = 0.017. Accuracy did not vary by Model gender, F(1,2567) = 0.32, *p* = 0.571, nor was there a Rater × Model interaction, F(1,2567) = 0.93, *p* = 0.336.

### 2. Intensity Ratings

An average intensity rating was computed for each image, and these ratings were used to compute an average intensity rating for each expression (Table 2). A one-way ANOVA with factor of Expression (Angry vs. Content vs. Disgusted vs. Afraid vs. Happy vs. Neutral vs. Sad vs. Surprised) revealed a significant main effect, F(7,1277) = 591.38, *p*<0.001. The Neutral images were rated least intense (1.26, SD = 0.19) and the Surprised images were rated most intense (4.11, SD = 0.57). Bonferroni pairwise comparisons indicated that all mean intensity ratings were different from each other except Disgusted (mean = 3.87, SD = 0.43) and Happy (mean = 3.71, SD = 0.44), and Happy and Afraid (mean = 3.64, SD = 0.58). A 2×2 ANOVA with factors of Rater (Male vs. Female) and Model (Male vs. Female) indicated that overall Female raters rated the images as more intense (mean = 3.35, SD = 1.00) than Male raters (mean = 3.11, SD = 0.97), F(1,2567) = 38.35, *p*<0.001, but there was no main effect of Model gender, F(1,2567) = 0.00, *p* = 0.970, nor was there a Rater × Model interaction, F(1,2567) = 0.56, *p* = 0.453.

**Table 2:** Mean (standard deviation) intensity ratings for each facial expression from the top 40 male and top 40 female models.

Expression	Intensity rating
Neutral	1.26 (0.19)
Content	2.76 (0.41)
Sad	3.20 (0.45)
Angry	3.37 (0.56)
Afraid	3.64 (0.58)
Happy	3.71 (0.44)
Disgusted	3.87 (0.43)
Surprised	4.11 (0.57)

1 = low intensity, 5 = high intensity.

### 3. Age Ratings

The final 80 models ranged in age from 6–16 years (mean = 9.84, SD = 2.33). Age estimates from raters were used to compute a mean age estimate for each model (Table 1). A paired t-test revealed that the mean estimated age of the models (mean = 9.23, SD = 2.27) was significantly younger than their actual mean age, t(79) = 3.51, p<0.001.

We also computed the difference between the mean estimate score and the true age for each model. The absolute value of the difference scores was used to compute a mean difference between estimated age and true age of all models. This mean difference was 1.36 years, and the range of deviations was –3.32 to +3.15 years. Using these absolute difference scores, we performed a 2×2 ANOVA with factors of Rater (Male vs. Female) and Model (Male vs. Female) and determined that there was no difference in the accuracy of age ratings by Rater gender, F(1,159) = 0.26, *p* = 0.609, or Model gender, F(1,159) = 1.15, *p* = 0.285, and no Rater by Model interaction, F(1,159) = 1.22, *p* = 0.271.

Finally, to assess inter-rater reliability for age estimates, we performed 10 separate two-way mixed Intraclass Correlations (one for each group of models, see Table 3). Four raters did not provide age estimates for all models and so were excluded from this analysis. All average measure Intraclass Correlation Coefficients (ICCs) were significant (all *ps*<0.001) and ranged from 0.93–0.99, indicating strong agreement for age estimates in all groups.

**Table 3:** Intraclass correlations for age ratings for each group of images for top 40 male and top 40 female models.

Group	Intraclass Correlation Coefficient	*p*
Males		
1	0.973	<0.001
2	0.959	<0.001
3	**0.934**	<0.001
4	0.979	<0.001
5	**0.990**	<0.001
Females		
1	0.982	<0.001
2	0.980	<0.001
3	0.970	<0.001
4	0.978	<0.001
5	0.989	<0.001

Largest and smallest correlations are indicated in bold.

## Discussion

Here we present the Dartmouth Database of Children’s Faces, a well-controlled database of faces of 40 male and 40 female Caucasian children. All faces were assessed by at least 20 raters for facial expression identifiability and intensity, and perceived age. There was good agreement between rater-chosen expressions and intended expressions. Consistent with other databases, Happy was most accurately identified [5], [7]–[9], [14]–[16] and Afraid was least accurately identified [7]–[9], [14], [16]. As in Ekman and Friesen’s Pictures of Facial Affect [8], [9], Afraid was most often confused with Surprised. Only a small percentage of images were identified as “None”, meaning that most facial expressions could be classified as one of the target expressions. Surprised was the most intense facial expression, whereas Neutral was the least intense. Happy was rated as more intense than Content, indicating that intensity ratings were meaningful. Raters were able to estimate the age of the models within a little over a year of their true age and showed strong agreement in age estimates. Consistent with previous findings of gender differences in face tasks [13], [17], [18], female raters were more accurate than male raters at identifying facial expressions. Female raters also rated facial expressions as more intense overall. Taken together, these findings support the validity of the expressions, and also provide information that will allow researchers to choose particular intensities of the expressions and perceived ages.

We sought to develop a database of images of children’s faces that would be of use to researchers from a variety of fields. One area of research that may particularly benefit from this database is the own-age bias: the effect where individuals are better able to identify faces of people their own age [8], [14], [16]. While this effect has important implications for stimulus selection in face perception studies, the perception of own- versus other-age faces can be influenced by personal characteristics of the observer (e.g. experience with own- or other-age faces [1]–[3], [19]–[23]). Although most research on the own-age bias has focused on identity recognition (but see [24], [25]), one interesting question is whether the own-age bias extends to expression identification. Our database is ideally suited to facilitate studies designed to answer this question. Investigations such as these, on the interaction between stimulus and observer age, provide important information about the development of normal face recognition and support evidence from cross-species [26], [27], and own- versus other-race studies [28]–[30] that suggest that a range of facial characteristics impact face perception. Given these considerations, it is worth restating that the image ratings provided here are from adult raters and that rater and model age may interact. Again, this is an interesting question for future research.

Although a single database will not satisfy the needs of every research study, our primary goal was to create a database that varied facial identity, gender, and expression, while minimizing variation in extra-facial features that could be used to distinguish individuals (e.g. jewelry, glasses, etc., but also skin color). While we chose to restrict our database to Caucasian children only, including children of different ethnicities would provide an avenue for answering additional research questions, such as those regarding other-race effects [29], [31], and the interaction between the perception of race and age. The acquisition of images of children of different ethnicities was beyond the scope of this study, but this is undoubtedly a desirable addition for future image sets.

In general, findings on the intersection between participant and stimulus emphasize the importance of using a broad range of well-controlled stimuli in vision research. Given the disparity in access to adult versus children face stimuli, we hope that our freely available database of children’s faces will help address this issue and fill the current void in stimulus databases, providing a useful tool for future research in a variety of areas.

## Supporting Information

Table S1 **Image ratings.** Image ratings for all images included in the Dartmouth Database of Children’s Faces. Images are listed by model number, file name, and intended expression. Rating is percent agreement between rater-chosen expression and intended expression.
(PDF)
