# Supplementary material for: The Dartmouth Database of Children’s Faces: Acquisition and Validation of a New Face Stimulus Set
Source: PLoS One. 2013 Nov 14;8(11):e79131. doi: 10.1371/journal.pone.0079131 (PMC3828408; doi:10.1371/journal.pone.0079131)
Supplement: Table S1 — Image ratings. Image ratings for all images included in the Dartmouth Database of Children’s Faces. Images are listed by model number, file name, and intended expression. Rating is percent agreement between rater-chosen expression and intended expression. (PDF) [file pone.0079131.s001.pdf]

Table S1:
Image ratings

Males			
**Model ID**	**Image ID**	**Intended Expression**	**Rating (%)**
**4**	04_13y_surprise_front2	Surprised	95.0
	04_13y_surprise	Surprised	90.0
	04_13yoM_Angry_front2	Angry	80.0
	04_13yoM_Angry	Angry	65.0
	04_13yoM_disgust_front2	Disgusted	35.0
	04_13yoM_disgust	Disgusted	55.0
	04_13yoM_Fear_front2	Afraid	55.0
	04_13yoM_Fear	Afraid	70.0
	04_13yoM	Neutral	95.0
	04_13yoM_front2	Neutral	95.0
	04_13yoM_HappyNoTeeth_front2	Content	100.0
	04_13yoM_HappyNoTeeth	Content	100.0
	04_13yoM_HappyTeeth_front2	Happy	100.0
	04_13yoM_HappyTeeth	Happy	100.0
	04_13yom_sad	Sad	70.0
**10**	10_9y_surprise_front2	Surprised	90.0
	10_9y_surprise	Surprised	85.0
	10_9yoM_Angry_front2	Angry	75.0
	10_9yoM_Angry	Angry	40.0
	10_9yoM_disgust_front2	Disgusted	90.0
	10_9yoM_disgust	Disgusted	90.0
	10_9yoM_Fear_front2	Afraid	15.0
	10_9yoM_Fear	Afraid	35.0
	10_9yoM	Neutral	100.0
	10_9yoM_front2	Neutral	90.0
	10_9yoM_HappyNoTeeth_front2	Content	100.0
	10_9yoM_HappyNoTeeth	Content	90.0
	10_9yoM_HappyTeeth_front2	Happy	95.0
	10_9yoM_HappyTeeth	Happy	100.0
	10_9yom_sad_front2	Sad	20.0
	10_9yom_sad	Sad	55.0
**11**	11_10y_surprise_front2	Surprised	85.0
	11_10y_surprise	Surprised	95.0
	11_10yoM_Angry_front2	Angry	10.0
	11_10yoM_Angry	Angry	20.0
	11_10yoM_disgust_front2	Disgusted	60.0
	11_10yoM_disgust	Disgusted	75.0
	11_10yoM_Fear_front2	Afraid	0.0
	11_10yoM_Fear	Afraid	5.0
	11_10yoM	Neutral	90.0
	11_10yoM_front2	Neutral	95.0
	11_10yoM_HappyNoTeeth_front2	Content	100.0

Females			
**Model ID**	**Image ID**	**Intended Expression**	**Rating (%)**
**13**	13_11yoF_Angry_front1	Angry	70.0
	13_11yoF_Angry_front2	Angry	70.0
	13_11yoF_Disgust_front1	Disgusted	65.0
	13_11yoF_Disgust_front2	Disgusted	75.0
	13_11yoF_Fear_front1	Afraid	75.0
	13_11yoF_Fear_front2	Afraid	65.0
	13_11yoF_front1	Neutral	80.0
	13_11yoF_front2	Neutral	45.0
	13_11yoF_HappyNoTeeth_front1	Content	95.0
	13_11yoF_HappyNoTeeth_front2	Content	90.0
	13_11yoF_HappyTeeth_front1	Happy	100.0
	13_11yoF_HappyTeeth_front2	Happy	95.0
	13_11yoF_Sad_front1	Sad	75.0
	13_11yoF_Sad_front2	Sad	85.0
	13_11yoF_Surprise_front1	Surprised	80.0
	13_11yoF_Surprise_front2	Surprised	75.0
**16**	16_11yoF_Angry_front1	Angry	30.0
	16_11yoF_Angry_front2	Angry	75.0
	16_11yoF_Disgust_front1	Disgusted	55.0
	16_11yoF_Disgust_front2	Disgusted	80.0
	16_11yoF_Fear_front1	Afraid	75.0
	16_11yoF_Fear_front2	Afraid	70.0
	16_11yoF_front1	Neutral	90.0
	16_11yoF_front2	Neutral	80.0
	16_11yoF_HappyNoTeeth_front1	Content	95.0
	16_11yoF_HappyNoTeeth_front2	Content	95.0
	16_11yoF_HappyTeeth_front1	Happy	95.0
	16_11yoF_HappyTeeth_front2	Happy	95.0
	16_11yoF_Sad_front1	Sad	50.0
	16_11yoF_Sad_front2	Sad	40.0
	16_11yoF_Surprise_front1	Surprised	65.0
	16_11yoF_Surprise_front2	Surprised	65.0
**22**	22_9yoF_Angry_front1	Angry	50.0
	22_9yoF_Angry_front2	Angry	90.0
	22_9yoF_Disgust_front1	Disgusted	15.0
	22_9yoF_Disgust_front2	Disgusted	50.0
	22_9yoF_Fear_front1	Afraid	10.0
	22_9yoF_Fear_front2	Afraid	30.0
	22_9yoF_front1	Neutral	90.0
	22_9yoF_front2	Neutral	85.0
	22_9yoF_HappyNoTeeth_front1	Content	90.0
	22_9yoF_HappyNoTeeth_front2	Content	85.0

Males			
**Model ID**	**Image ID**	**Intended Expression**	**Rating (%)**
**11 cont…**	11_10yoM_HappyNoTeeth	Content	100.0
	11_10yoM_HappyTeeth_front2	Happy	100.0
	11_10yoM_HappyTeeth	Happy	100.0
	11_10yom_sad_front2	Sad	80.0
	11_10yom_sad_front3	Sad	80.0
	11_10yom_sad_front4	Sad	85.0
	11_10yom_sad	Sad	85.0
**27**	27_9y_surprise_front2	Surprised	90.0
	27_9y_surprise	Surprised	85.0
	27_9yoM_Angry_front2	Angry	65.0
	27_9yoM_Angry	Angry	65.0
	27_9yoM_disgust_front2	Disgusted	90.0
	27_9yoM_disgust	Disgusted	90.0
	27_9yoM_Fear_front2	Afraid	45.0
	27_9yoM_Fear	Afraid	30.0
	27	Neutral	80.0
	27_9yoM_front2	Neutral	65.0
	27_9yoM_HappyNoTeeth_front2	Content	60.0
	27_9yoM_HappyNoTeeth	Content	80.0
	27_9yoM_HappyTeeth_front2	Happy	100.0
	27_9yoM_HappyTeeth	Happy	100.0
	27_9yom_sad_front2	Sad	90.0
	27_9yom_sad	Sad	100.0
**28**	28_11y_surprise_front2	Surprised	95.0
	28_11y_surprise	Surprised	95.0
	28_11yoM_Angry_front2	Angry	20.0
	28_11yoM_Angry	Angry	35.0
	28_11yoM_disgust_front2	Disgusted	90.0
	28_11yoM_disgust_front3	Disgusted	90.0
	28_11yoM_disgust	Disgusted	60.0
	28_11yoM_Fear_front2	Afraid	0.0
	28_11yoM_Fear	Afraid	5.0
	28_11yoM	Neutral	85.0
	28_11yoM_front2	Neutral	95.0
	28_11yoM_HappyNoTeeth_front2	Content	95.0
	28_11yoM_HappyNoTeeth	Content	85.0
	28_11yoM_HappyTeeth_front2	Happy	100.0
	28_11yoM_HappyTeeth	Happy	100.0
	28_11yom_sad_front2	Sad	100.0
	28_11yom_sad	Sad	95.0
**29**	29_15y_surprise_front2	Surprised	90.0

Females			
**Model ID**	**Image ID**	**Intended Expression**	**Rating (%)**
**22 cont…**	22_9yoF_HappyTeeth_front1	Happy	100.0
	22_9yoF_HappyTeeth_front2	Happy	85.0
	22_9yoF_Sad_front1	Sad	90.0
	22_9yoF_Sad_front2	Sad	85.0
	22_9yoF_Surprise_front1	Surprised	95.0
	22_9yoF_Surprise_front2	Surprised	90.0
**25**	25_13yoF_Angry_front1	Angry	30.0
	25_13yoF_Angry_front2	Angry	50.0
	25_13yoF_Disgust_front1	Disgusted	90.0
	25_13yoF_Disgust_front2	Disgusted	75.0
	25_13yoF_Fear_front1	Afraid	60.0
	25_13yoF_Fear_front2	Afraid	45.0
	25_13yoF_front1	Neutral	85.0
	25_13yoF_front2	Neutral	75.0
	25_13yoF_HappyNoTeeth_front1	Content	50.0
	25_13yoF_HappyNoTeeth_front2	Content	60.0
	25_13yoF_HappyTeeth_front1	Happy	100.0
	25_13yoF_HappyTeeth_front2	Happy	95.0
	25_13yoF_Sad_front1	Sad	85.0
	25_13yoF_Sad_front2	Sad	80.0
	25_13yoF_Surprise_front1	Surprised	60.0
	25_13yoF_Surprise_front2	Surprised	85.0
**26**	26_7yoF_Angry_front1	Angry	70.0
	26_7yoF_Angry_front2	Angry	55.0
	26_7yoF_Disgust_front1	Disgusted	85.0
	26_7yoF_Disgust_front2	Disgusted	60.0
	26_7yoF_Fear_front1	Afraid	0.0
	26_7yoF_Fear_front2	Afraid	0.0
	26_7yoF_front1	Neutral	80.0
	26_7yoF_front2	Neutral	85.0
	26_7yoF_HappyNoTeeth_front1	Content	95.0
	26_7yoF_HappyNoTeeth_front2	Content	100.0
	26_7yoF_HappyTeeth_front1	Happy	95.0
	26_7yoF_HappyTeeth_front2	Happy	95.0
	26_7yoF_Sad_front1	Sad	100.0
	26_7yoF_Sad_front2	Sad	90.0
	26_7yoF_Surprise_front1	Surprised	90.0
	26_7yoF_Surprise_front2	Surprised	80.0
**32**	32_6yoF_Angry_front1	Angry	90.0
	32_6yoF_Angry_front2	Angry	95.0
	32_6yoF_Disgust_front1	Disgusted	85.0

Males			
Model ID	Image ID	Intended Expression	Rating (%)
29 cont…	29_15y_surprise	Surprised	95.0
	29_15yoM_Angry_front2	Angry	80.0
	29_15yoM_Angry_front3	Angry	85.0
	29_15yoM_Angry	Angry	75.0
	29_15yoM_disgust_front2	Disgusted	95.0
	29_15yoM_disgust_front3	Disgusted	95.0
	29_15yoM_disgust	Disgusted	70.0
	29_15yoM_Fear_front2	Afraid	60.0
	29_15yoM_Fear	Afraid	60.0
	29_15yoM	Neutral	100.0
	29_15yoM_front2	Neutral	100.0
	29_15yoM_HappyNoTeeth_front2	Content	100.0
	29_15yoM_HappyNoTeeth	Content	95.0
	29_15yoM_HappyTeeth_front2	Happy	100.0
	29_15yoM_HappyTeeth	Happy	100.0
	29_15yom_sad_front2	Sad	80.0
	29_15yom_sad_front3	Sad	95.0
	29_15yom_sad	Sad	65.0
36	36_11y_surprise_front2	Surprised	90.0
	36_11y_surprise	Surprised	95.0
	36_11yoM_Angry_front2	Angry	90.0
	36_11yoM_Angry	Angry	95.0
	36_11yoM_disgust_front2	Disgusted	70.0
	36_11yoM_disgust	Disgusted	75.0
	36_11yoM_Fear_front2	Afraid	30.0
	36_11yoM_Fear	Afraid	50.0
	36_11yoM	Neutral	85.0
	36_11yoM_front2	Neutral	95.0
	36_11yoM_HappyNoTeeth_front2	Content	50.0
	36_11yoM_HappyNoTeeth	Content	70.0
	36_11yoM_HappyTeeth_front2	Happy	95.0
	36_11yoM_HappyTeeth	Happy	100.0
	36_11yom_sad_front2	Sad	95.0
	36_11yom_sad	Sad	100.0
37	37_8y_surprise_front2	Surprised	90.0
	37_8y_surprise	Surprised	100.0
	37_8yoM_Angry_front2	Angry	80.0
	37_8yoM_Angry	Angry	65.0
	37_8yoM_disgust_front2	Disgusted	90.0
	37_8yoM_disgust	Disgusted	90.0
	37_8yoM_Fear_front2	Afraid	50.0

Females			
Model ID	Image ID	Intended Expression	Rating (%)
32 cont…	32_6yoF_Disgust_front2	Disgusted	35.0
	32_6yoF_Fear_front1	Afraid	0.0
	32_6yoF_Fear_front2	Afraid	70.0
	32_6yoF_front1	Neutral	45.0
	32_6yoF_front2	Neutral	75.0
	32_6yoF_HappyNoTeeth_front1	Content	95.0
	32_6yoF_HappyNoTeeth_front2	Content	95.0
	32_6yoF_HappyTeeth_front1	Happy	100.0
	32_6yoF_HappyTeeth_front2	Happy	100.0
	32_6yoF_Sad_front1	Sad	100.0
	32_6yoF_Sad_front2	Sad	100.0
	32_6yoF_Surprise_front1	Surprised	95.0
	32_6yoF_Surprise_front2	Surprised	95.0
33	33_6yoF_Angry_front	Angry	20.0
	33_6yoF_Disgust_front1	Disgusted	80.0
	33_6yoF_Disgust_front2	Disgusted	60.0
	33_6yoF_Fear_front1	Afraid	75.0
	33_6yoF_Fear_front2	Afraid	30.0
	33_6yoF_front1	Neutral	95.0
	33_6yoF_front2	Neutral	100.0
	33_6yoF_HappyNoTeeth_front1	Content	70.0
	33_6yoF_HappyNoTeeth_front2	Content	85.0
	33_6yoF_HappyTeeth_front1	Happy	100.0
	33_6yoF_HappyTeeth_front2	Happy	95.0
	33_6yoF_Sad_front1	Sad	100.0
	33_6yoF_Sad_front2	Sad	90.0
	33_6yoF_Surprise_front1	Surprised	100.0
	33_6yoF_Surprise_front2	Surprised	100.0
45	45_7yoF_Angry_front1	Angry	60.0
	45_7yoF_Angry_front2	Angry	60.0
	45_7yoF_Disgust_front1	Disgusted	85.0
	45_7yoF_Disgust_front2	Disgusted	25.0
	45_7yoF_Fear_front1	Afraid	45.0
	45_7yoF_Fear_front2	Afraid	35.0
	45_7yoF_front1	Neutral	100.0
	45_7yoF_front2	Neutral	95.0
	45_7yoF_HappyNoTeeth_front1	Content	100.0
	45_7yoF_HappyNoTeeth_front2	Content	75.0
	45_7yoF_HappyTeeth_front1	Happy	100.0
	45_7yoF_HappyTeeth_front2	Happy	100.0
	45_7yoF_Sad_front1	Sad	95.0

Males			
Model ID	Image ID	Intended Expression	Rating (%)
37 cont…	37_8yoM_Fear	Afraid	35.0
	37_8yoM	Neutral	95.0
	37_8yoM_front3	Neutral	100.0
	37_8yoM_HappyNoTeeth_front2	Content	100.0
	37_8yoM_HappyNoTeeth	Content	100.0
	37_8yoM_HappyTeeth_front2	Happy	100.0
	37_8yoM_HappyTeeth	Happy	85.0
	37_8yom_sad_front2	Sad	100.0
	37_8yom_sad	Sad	85.0
38	38_10y_surprise_front2	Surprised	90.0
	38_10y_surprise	Surprised	90.0
	38_10yoM_Angry_front2	Angry	100.0
	38_10yoM_Angry	Angry	90.0
	38_10yoM_disgust_front2	Disgusted	50.0
	38_10yoM_disgust	Disgusted	75.0
	38_10yoM_Fear_front2	Afraid	45.0
	38_10yoM_Fear	Afraid	60.0
	38_10yoM	Neutral	95.0
	38_10yoM_front2	Neutral	85.0
	38_10yoM_HappyNoTeeth_front2	Content	85.0
	38_10yoM_HappyNoTeeth	Content	95.0
	38_10yoM_HappyTeeth_front2	Happy	100.0
	38_10yoM_HappyTeeth	Happy	100.0
	38_10yom_sad_front2	Sad	100.0
	38_10yom_sad	Sad	90.0
40	40_12y_surprise_front2	Surprised	75.0
	40_12y_surprise	Surprised	75.0
	40_12yoM_Angry_front2	Angry	95.0
	40_12yoM_Angry	Angry	90.0
	40_12yoM_disgust_front2	Disgusted	80.0
	40_12yoM_disgust	Disgusted	55.0
	40_12yoM_Fear_front2	Afraid	45.0
	40_12yoM_Fear	Afraid	45.0
	40_12yoM	Neutral	90.0
	40_12yoM_front2	Neutral	95.0
	40_12yoM_HappyNoTeeth_front2	Content	100.0
	40_12yoM_HappyNoTeeth	Content	90.0
	40_12yoM_HappyTeeth_front2	Happy	95.0
	40_12yoM_HappyTeeth	Happy	80.0
	40_12yom_sad_front2	Sad	100.0
	40_12yom_sad	Sad	90.0

Females			
Model ID	Image ID	Intended Expression	Rating (%)
45 cont…	45_7yoF_Sad_front2	Sad	85.0
	45_7yoF_Surprise_front1	Surprised	75.0
	45_7yoF_Surprise_front2	Surprised	80.0
52	52_10yoF_Angry_front1	Angry	100.0
	52_10yoF_Angry_front2	Angry	95.0
	52_10yoF_Disgust_front1	Disgusted	75.0
	52_10yoF_Disgust_front2	Disgusted	50.0
	52_10yoF_Fear_front1	Afraid	45.0
	52_10yoF_Fear_front2	Afraid	40.0
	52_10yoF_front1	Neutral	90.0
	52_10yoF_front2	Neutral	100.0
	52_10yoF_HappyNoTeeth_front1	Content	85.0
	52_10yoF_HappyNoTeeth_front2	Content	75.0
	52_10yoF_HappyTeeth_front1	Happy	100.0
	52_10yoF_HappyTeeth_front2	Happy	100.0
	52_10yoF_Sad_front1	Sad	100.0
	52_10yoF_Sad_front2	Sad	55.0
	52_10yoF_Surprise_front1	Surprised	80.0
	52_10yoF_Surprise_front2	Surprised	80.0
55	55_10yoF_Angry_front1	Angry	55.0
	55_10yoF_Angry_front2	Angry	65.0
	55_10yoF_Disgust_front1	Disgusted	90.0
	55_10yoF_Disgust_front2	Disgusted	75.0
	55_10yoF_Fear_front1	Afraid	60.0
	55_10yoF_Fear_front2	Afraid	45.0
	55_10yoF_front1	Neutral	90.0
	55_10yoF_front2	Neutral	100.0
	55_10yoF_HappyNoTeeth_front1	Content	70.0
	55_10yoF_HappyNoTeeth_front2	Content	70.0
	55_10yoF_HappyTeeth_front1	Happy	95.0
	55_10yoF_HappyTeeth_front2	Happy	100.0
	55_10yoF_Sad_front1	Sad	30.0
	55_10yoF_Sad_front2	Sad	50.0
	55_10yoF_Surprise_front1	Surprised	80.0
	55_10yoF_Surprise_front2	Surprised	95.0
57	57_9yoF_Angry_front1	Angry	70.0
	57_9yoF_Angry_front2	Angry	90.0
	57_9yoF_Disgust_front1	Disgusted	45.0
	57_9yoF_Disgust_front2	Disgusted	40.0
	57_9yoF_Fear_front1	Afraid	60.0
	57_9yoF_Fear_front2	Afraid	50.0

Males			
Model ID	Image ID	Intended Expression	Rating (%)
**41**	41_8y_surprise_front2	Surprised	95.0
	41_8y_surprise	Surprised	90.0
	41_8yoM_Angry_front2	Angry	20.0
	41_8yoM_Angry	Angry	50.0
	41_8yoM_disgust_front2	Disgusted	100.0
	41_8yoM_disgust	Disgusted	85.0
	41_8yoM_Fear_front2	Afraid	40.0
	41_8yoM_Fear	Afraid	25.0
	41_8yoM	Neutral	95.0
	41_8yoM_front2	Neutral	90.0
	41_8yoM_HappyNoTeeth_front2	Content	50.0
	41_8yoM_HappyNoTeeth	Content	70.0
	41_8yoM_HappyTeeth_front2	Happy	100.0
	41_8yoM_HappyTeeth	Happy	100.0
	41_8yom_sad_front2	Sad	100.0
	41_8yom_sad	Sad	95.0
**44**	44_11y_surprise_front2	Surprised	35.0
	44_11y_surprise	Surprised	40.0
	44_11yoM_Angry_front2	Angry	70.0
	44_11yoM_Angry	Angry	85.0
	44_11yoM_disgust_front2	Disgusted	45.0
	44_11yoM_disgust	Disgusted	45.0
	44_11yoM_Fear_front2	Afraid	50.0
	44_11yoM_Fear	Afraid	60.0
	44_11yoM	Neutral	75.0
	44_11yoM_front2	Neutral	100.0
	44_11yoM_HappyNoTeeth_front2	Content	100.0
	44_11yoM_HappyNoTeeth	Content	100.0
	44_11yoM_HappyTeeth_front2	Happy	90.0
	44_11yoM_HappyTeeth	Happy	100.0
	44_11yom_sad_front2	Sad	100.0
	44_11yom_sad	Sad	90.0
**48**	48_11y_surprise_front2	Surprised	90.0
	48_11y_surprise	Surprised	85.0
	48_11yoM_Angry_front2	Angry	0.0
	48_11yoM_Angry	Angry	15.0
	48_11yoM_disgust_front2	Disgusted	40.0
	48_11yoM_disgust	Disgusted	35.0
	48_11yoM_Fear_front2	Afraid	55.0
	48_11yoM_Fear	Afraid	60.0
	48_11yoM	Neutral	100.0

Females			
Model ID	Image ID	Intended Expression	Rating (%)
**57 cont…**	57_9yoF_front1	Neutral	95.0
	57_9yoF_front2	Neutral	90.0
	57_9yoF_HappyNoTeeth_front1	Content	95.0
	57_9yoF_HappyNoTeeth_front2	Content	100.0
	57_9yoF_HappyTeeth_front1	Happy	100.0
	57_9yoF_HappyTeeth_front2	Happy	100.0
	57_9yoF_Sad_front1	Sad	50.0
	57_9yoF_Sad_front2	Sad	45.0
	57_9yoF_Surprise_front1	Surprised	80.0
	57_9yoF_Surprise_front2	Surprised	95.0
**61**	61_8yoF_Angry_front1	Angry	75.0
	61_8yoF_Angry_front2	Angry	75.0
	61_8yoF_Disgust_front1	Disgusted	10.0
	61_8yoF_Disgust_front2	Disgusted	10.0
	61_8yoF_Fear_front1	Afraid	30.0
	61_8yoF_Fear_front2	Afraid	50.0
	61_8yoF_front1	Neutral	85.0
	61_8yoF_front2	Neutral	85.0
	61_8yoF_HappyNoTeeth_front1	Content	100.0
	61_8yoF_HappyNoTeeth_front2	Content	95.0
	61_8yoF_HappyTeeth_front1	Happy	100.0
	61_8yoF_HappyTeeth_front2	Happy	95.0
	61_8yoF_Sad_front1	Sad	100.0
	61_8yoF_Sad_front2	Sad	95.0
	61_8yoF_Surprise_front1	Surprised	95.0
	61_8yoF_Surprise_front2	Surprised	95.0
**62**	62_10yoF_Angry_front1	Angry	55.0
	62_10yoF_Angry_front2	Angry	60.0
	62_10yoF_Disgust_front1	Disgusted	95.0
	62_10yoF_Disgust_front2	Disgusted	90.0
	62_10yoF_Fear_front1	Afraid	45.0
	62_10yoF_Fear_front2	Afraid	60.0
	62_10yoF_front1	Neutral	85.0
	62_10yoF_front2	Neutral	90.0
	62_10yoF_HappyNoTeeth_front1	Content	95.0
	62_10yoF_HappyNoTeeth_front2	Content	85.0
	62_10yoF_HappyTeeth_front1	Happy	90.0
	62_10yoF_HappyTeeth_front2	Happy	95.0
	62_10yoF_Sad_front1	Sad	90.0
	62_10yoF_Sad_front2	Sad	95.0
	62_10yoF_Surprise_front1	Surprised	70.0

Males			
**Model ID**	**Image ID**	**Intended Expression**	**Rating (%)**
**48 cont…**	48_11yoM_front2	Neutral	95.0
	48_11yoM_HappyNoTeeth_front2	Content	95.0
	48_11yoM_HappyNoTeeth	Content	100.0
	48_11yoM_HappyTeeth_front2	Happy	100.0
	48_11yoM_HappyTeeth	Happy	100.0
	48_11yom_sad_front2	Sad	95.0
	48_11yom_sad	Sad	95.0
**49**	49_8y_surprise_front2	Surprised	85.0
	49_8y_surprise	Surprised	90.0
	49_8yoM_Angry_front2	Angry	60.0
	49_8yoM_Angry	Angry	55.0
	49_8yoM_disgust_front2	Disgusted	35.0
	49_8yoM_disgust	Disgusted	30.0
	49_8yoM_Fear_front2	Afraid	55.0
	49_8yoM_Fear	Afraid	60.0
	49_8yoM	Neutral	95.0
	49_8yoM_front2	Neutral	95.0
	49_8yoM_HappyNoTeeth_front2	Content	100.0
	49_8yoM_HappyNoTeeth	Content	100.0
	49_8yoM_HappyTeeth_front2	Happy	100.0
	49_8yoM_HappyTeeth	Happy	95.0
	49_8yom_sad_front2	Sad	90.0
	49_8yom_sad	Sad	85.0
**51**	51_11y_surprise_front2	Surprised	40.0
	51_11y_surprise	Surprised	55.0
	51_11yoM_Angry_front2	Angry	75.0
	51_11yoM_Angry	Angry	75.0
	51_11yoM_disgust_front2	Disgusted	85.0
	51_11yoM_disgust	Disgusted	80.0
	51_11yoM_Fear_front2	Afraid	90.0
	51_11yoM_Fear	Afraid	70.0
	51_11yoM	Neutral	90.0
	51_11yoM_front2	Neutral	90.0
	51_11yoM_HappyNoTeeth_front2	Content	100.0
	51_11yoM_HappyNoTeeth	Content	100.0
	51_11yoM_HappyTeeth_front2	Happy	100.0
	51_11yoM_HappyTeeth	Happy	100.0
	51_11yom_sad_front2	Sad	100.0
	51_11yom_sad	Sad	75.0
**53**	53_8y_surprise_front2	Surprised	55.0
	53_8y_surprise	Surprised	50.0

Females			
**Model ID**	**Image ID**	**Intended Expression**	**Rating (%)**
**62 cont…**	62_10yoF_Surprise_front2	Surprised	75.0
**64**	64_10yoF_Angry_front1	Angry	90.0
	64_10yoF_Angry_front2	Angry	95.0
	64_10yoF_Disgust_front1	Disgusted	85.0
	64_10yoF_Disgust_front2	Disgusted	95.0
	64_10yoF_Fear_front1	Afraid	30.0
	64_10yoF_Fear_front2	Afraid	15.0
	64_10yoF_front1	Neutral	75.0
	64_10yoF_front2	Neutral	85.0
	64_10yoF_HappyNoTeeth_front1	Content	100.0
	64_10yoF_HappyNoTeeth_front2	Content	95.0
	64_10yoF_HappyTeeth_front1	Happy	90.0
	64_10yoF_HappyTeeth_front2	Happy	95.0
	64_10yoF_Sad_front1	Sad	95.0
	64_10yoF_Sad_front2	Sad	95.0
	64_10yoF_Surprise_front1	Surprised	80.0
	64_10yoF_Surprise_front2	Surprised	70.0
**65**	65_8yoF_Angry_front1	Angry	65.0
	65_8yoF_Angry_front2	Angry	70.0
	65_8yoF_Disgust_front1	Disgusted	85.0
	65_8yoF_Disgust_front2	Disgusted	80.0
	65_8yoF_Fear_front1	Afraid	50.0
	65_8yoF_Fear_front2	Afraid	45.0
	65_8yoF_front1	Neutral	65.0
	65_8yoF_front2	Neutral	95.0
	65_8yoF_HappyNoTeeth_front1	Content	90.0
	65_8yoF_HappyNoTeeth_front2	Content	100.0
	65_8yoF_HappyTeeth_front1	Happy	95.0
	65_8yoF_HappyTeeth_front2	Happy	95.0
	65_8yoF_Sad_front1	Sad	95.0
	65_8yoF_Sad_front2	Sad	70.0
	65_8yoF_Surprise_front1	Surprised	80.0
	65_8yoF_Surprise_front2	Surprised	80.0
**69**	69_7yoF_Angry_front1	Angry	65.0
	69_7yoF_Angry_front2	Angry	60.0
	69_7yoF_Disgust_front1	Disgusted	85.0
	69_7yoF_Disgust_front2	Disgusted	80.0
	69_7yoF_Fear_front1	Afraid	30.0
	69_7yoF_Fear_front2	Afraid	5.0
	69_7yoF_front1	Neutral	90.0
	69_7yoF_front2	Neutral	90.0

Males			
Model ID	Image ID	Intended Expression	Rating (%)
53 cont…	53_8yoM_Angry_front2	Angry	35.0
	53_8yoM_Angry	Angry	5.0
	53_8yoM_disgust_front2	Disgusted	90.0
	53_8yoM_disgust	Disgusted	85.0
	53_8yoM_Fear_front2	Afraid	65.0
	53_8yoM_Fear	Afraid	70.0
	53_8yoM	Neutral	90.0
	53_8yoM_front2	Neutral	90.0
	53_8yoM_HappyNoTeeth_front2	Content	95.0
	53_8yoM_HappyNoTeeth	Content	100.0
	53_8yoM_HappyTeeth_front2	Happy	100.0
	53_8yoM_HappyTeeth	Happy	100.0
	53_8yom_sad_front2	Sad	90.0
	53_8yom_sad	Sad	100.0
56	56_8y_surprise_front2	Surprised	95.0
	56_8y_surprise	Surprised	90.0
	56_8yoM_Angry_front2	Angry	85.0
	56_8yoM_Angry	Angry	100.0
	56_8yoM_disgust_front2	Disgusted	50.0
	56_8yoM_disgust	Disgusted	80.0
	56_8yoM_Fear_front2	Afraid	45.0
	56_8yoM_Fear	Afraid	55.0
	56_8yoM	Neutral	95.0
	56_8yoM_front2	Neutral	90.0
	56_8yoM_HappyNoTeeth_front2	Content	35.0
	56_8yoM_HappyNoTeeth	Content	50.0
	56_8yoM_HappyTeeth_front2	Happy	100.0
	56_8yoM_HappyTeeth	Happy	100.0
	56_8yom_sad_front2	Sad	90.0
	56_8yom_sad	Sad	85.0
58	58_8y_surprise_front2	Surprised	45.0
	58_8y_surprise	Surprised	55.0
	58_8yoM_Angry_front2	Angry	60.0
	58_8yoM_Angry	Angry	65.0
	58_8yoM_disgust_front2	Disgusted	75.0
	58_8yoM_disgust	Disgusted	75.0
	58_8yoM_Fear_front2	Afraid	30.0
	58_8yoM_Fear	Afraid	40.0
	58_8yoM	Neutral	85.0
	58_8yoM_front2	Neutral	80.0
	58_8yoM_HappyNoTeeth_front2	Content	95.0

Females			
Model ID	Image ID	Intended Expression	Rating (%)
69 cont…	69_7yoF_HappyNoTeeth_front1	Content	100.0
	69_7yoF_HappyNoTeeth_front2	Content	95.0
	69_7yoF_HappyTeeth_front1	Happy	100.0
	69_7yoF_HappyTeeth_front2	Happy	100.0
	69_7yoF_Sad_front1	Sad	100.0
	69_7yoF_Sad_front2	Sad	95.0
	69_7yoF_Surprise_front1	Surprised	70.0
	69_7yoF_Surprise_front2	Surprised	80.0
71	71_9yoF_Angry_front1	Angry	5.0
	71_9yoF_Angry_front2	Angry	15.0
	71_9yoF_Disgust_front1	Disgusted	85.0
	71_9yoF_Disgust_front2	Disgusted	80.0
	71_9yoF_Fear_front1	Afraid	55.0
	71_9yoF_Fear_front2	Afraid	80.0
	71_9yoF_front1	Neutral	90.0
	71_9yoF_front2	Neutral	95.0
	71_9yoF_HappyNoTeeth_front1	Content	100.0
	71_9yoF_HappyNoTeeth_front2	Content	100.0
	71_9yoF_HappyTeeth_front1	Happy	100.0
	71_9yoF_HappyTeeth_front2	Happy	100.0
	71_9yoF_Sad_front1	Sad	85.0
	71_9yoF_Sad_front2	Sad	90.0
	71_9yoF_Surprise_front1	Surprised	45.0
	71_9yoF_Surprise_front2	Surprised	55.0
74	74_8yoF_Angry_front1	Angry	85.0
	74_8yoF_Angry_front2	Angry	80.0
	74_8yoF_Disgust_front1	Disgusted	85.0
	74_8yoF_Disgust_front2	Disgusted	65.0
	74_8yoF_Fear_front1	Afraid	5.0
	74_8yoF_Fear_front2	Afraid	15.0
	74_8yoF_front1	Neutral	95.0
	74_8yoF_front2	Neutral	95.0
	74_8yoF_HappyNoTeeth_front1	Content	90.0
	74_8yoF_HappyNoTeeth_front2	Content	100.0
	74_8yoF_HappyTeeth_front1	Happy	95.0
	74_8yoF_HappyTeeth_front2	Happy	95.0
	74_8yoF_Sad_front1	Sad	55.0
	74_8yoF_Sad_front2	Sad	50.0
	74_8yoF_Surprise_front1	Surprised	85.0
	74_8yoF_Surprise_front2	Surprised	90.0
77	77_10yoF_Angry_front1	Angry	40.0

Males			
**Model ID**	**Image ID**	**Intended Expression**	**Rating (%)**
**58 cont…**	58_8yoM_HappyNoTeeth	Content	90.0
	58_8yoM_HappyTeeth_front2	Happy	100.0
	58_8yoM_HappyTeeth	Happy	95.0
	58_8yom_sad_front2	Sad	100.0
	58_8yom_sad	Sad	95.0
**59**	59_8y_surprise_front2	Surprised	80.0
	59_8y_surprise	Surprised	80.0
	59_8yoM_Angry_front2	Angry	70.0
	59_8yoM_Angry	Angry	55.0
	59_8yoM_disgust_front2	Disgusted	40.0
	59_8yoM_disgust	Disgusted	30.0
	59_8yoM_Fear_front2	Afraid	85.0
	59_8yoM_Fear	Afraid	85.0
	59_8yoM	Neutral	100.0
	59_8yoM_front2	Neutral	100.0
	59_8yoM_HappyNoTeeth_front2	Content	75.0
	59_8yoM_HappyNoTeeth	Content	90.0
	59_8yoM_HappyTeeth_front2	Happy	100.0
	59_8yoM_HappyTeeth	Happy	95.0
	59_8yom_sad_front2	Sad	100.0
	59_8yom_sad	Sad	100.0
**60**	60_7y_surprise_front2	Surprised	55.0
	60_7y_surprise	Surprised	70.0
	60_7yoM_Angry_front2	Angry	95.0
	60_7yoM_Angry	Angry	90.0
	60_7yoM_disgust_front2	Disgusted	75.0
	60_7yoM_disgust	Disgusted	80.0
	60_7yoM_Fear_front2	Afraid	75.0
	60_7yoM_Fear	Afraid	75.0
	60_7yoM	Neutral	95.0
	60_7yoM_front2	Neutral	95.0
	60_7yoM_HappyNoTeeth_front2	Content	85.0
	60_7yoM_HappyNoTeeth	Content	80.0
	60_7yoM_HappyTeeth_front2	Happy	90.0
	60_7yoM_HappyTeeth	Happy	100.0
	60_7yom_sad_front2	Sad	100.0
	60_7yom_sad	Sad	100.0
**63**	63_10y_surprise_front2	Surprised	80.0
	63_10y_surprise	Surprised	90.0
	63_10yoM_Angry_front2	Angry	70.0
	63_10yoM_Angry	Angry	65.0

Females			
**Model ID**	**Image ID**	**Intended Expression**	**Rating (%)**
**77 cont…**	77_10yoF_Angry_front2	Angry	50.0
	77_10yoF_Disgust_front1	Disgusted	85.0
	77_10yoF_Disgust_front2	Disgusted	85.0
	77_10yoF_Fear_front1	Afraid	50.0
	77_10yoF_Fear_front2	Afraid	75.0
	77_10yoF_front1	Neutral	95.0
	77_10yoF_front2	Neutral	95.0
	77_10yoF_HappyNoTeeth_front1	Content	100.0
	77_10yoF_HappyNoTeeth_front2	Content	100.0
	77_10yoF_HappyTeeth_front1	Happy	95.0
	77_10yoF_HappyTeeth_front2	Happy	100.0
	77_10yoF_Sad_front1	Sad	95.0
	77_10yoF_Sad_front2	Sad	100.0
	77_10yoF_Surprise_front1	Surprised	95.0
	77_10yoF_Surprise_front2	Surprised	100.0
**78**	78_9yoF_Angry_front1	Angry	85.0
	78_9yoF_Angry_front2	Angry	85.0
	78_9yoF_Disgust_front1	Disgusted	70.0
	78_9yoF_Disgust_front2	Disgusted	65.0
	78_9yoF_Fear_front1	Afraid	5.0
	78_9yoF_Fear_front2	Afraid	10.0
	78_9yoF_front1	Neutral	60.0
	78_9yoF_front2	Neutral	75.0
	78_9yoF_HappyNoTeeth_front1	Content	100.0
	78_9yoF_HappyNoTeeth_front2	Content	100.0
	78_9yoF_HappyTeeth_front1	Happy	95.0
	78_9yoF_HappyTeeth_front2	Happy	90.0
	78_9yoF_Sad_front1	Sad	85.0
	78_9yoF_Sad_front2	Sad	95.0
	78_9yoF_Surprise_front1	Surprised	90.0
	78_9yoF_Surprise_front2	Surprised	95.0
**88**	88_12yoF_Angry_front1	Angry	55.0
	88_12yoF_Angry_front2	Angry	70.0
	88_12yoF_Disgust_front1	Disgusted	65.0
	88_12yoF_Disgust_front2	Disgusted	70.0
	88_12yoF_Fear_front1	Afraid	10.0
	88_12yoF_Fear_front2	Afraid	20.0
	88_12yoF_front1	Neutral	95.0
	88_12yoF_front2	Neutral	95.0
	88_12yoF_HappyNoTeeth_front1	Content	100.0
	88_12yoF_HappyNoTeeth_front2	Content	100.0

Males			
**Model ID**	**Image ID**	**Intended Expression**	**Rating (%)**
**63 cont…**	63_10yoM_disgust_front2	Disgusted	65.0
	63_10yoM_disgust	Disgusted	65.0
	63_10yoM_Fear_front2	Afraid	35.0
	63_10yoM_Fear	Afraid	25.0
	63_10yoM	Neutral	95.0
	63_10yoM_front2	Neutral	95.0
	63_10yoM_HappyNoTeeth_front2	Content	75.0
	63_10yoM_HappyNoTeeth	Content	70.0
	63_10yoM_HappyTeeth_front2	Happy	100.0
	63_10yoM_HappyTeeth	Happy	100.0
	63_10yom_sad_front2	Sad	85.0
	63_10yom_sad	Sad	90.0
**66**	66_8y_surprise_front2	Surprised	75.0
	66_8y_surprise	Surprised	70.0
	66_8yoM_Angry_front2	Angry	20.0
	66_8yoM_Angry	Angry	25.0
	66_8yoM_disgust_front2	Disgusted	45.0
	66_8yoM_disgust	Disgusted	50.0
	66_8yoM_Fear_front2	Afraid	40.0
	66_8yoM_Fear	Afraid	65.0
	66_8yoM	Neutral	85.0
	66_8yoM_front2	Neutral	90.0
	66_8yoM_HappyNoTeeth_front2	Content	85.0
	66_8yoM_HappyNoTeeth	Content	100.0
	66_8yoM_HappyTeeth_front2	Happy	100.0
	66_8yoM_HappyTeeth	Happy	100.0
	66_8yom_sad_front2	Sad	90.0
	66_8yom_sad	Sad	80.0
**68**	68_9y_surprise_front2	Surprised	85.0
	68_9y_surprise	Surprised	90.0
	68_9yoM_Angry_front2	Angry	65.0
	68_9yoM_Angry	Angry	70.0
	68_9yoM_disgust_front2	Disgusted	85.0
	68_9yoM_disgust	Disgusted	25.0
	68_9yoM_Fear_front2	Afraid	50.0
	68_9yoM_Fear	Afraid	50.0
	68_9yoM	Neutral	100.0
	68_9yoM_front2	Neutral	100.0
	68_9yoM_HappyNoTeeth_front2	Content	95.0
	68_9yoM_HappyNoTeeth	Content	95.0
	68_9yoM_HappyTeeth_front2	Happy	95.0

Females			
**Model ID**	**Image ID**	**Intended Expression**	**Rating (%)**
**88 cont…**	88_12yoF_HappyTeeth_front1	Happy	95.0
	88_12yoF_HappyTeeth_front2	Happy	95.0
	88_12yoF_Sad_front1	Sad	85.0
	88_12yoF_Sad_front2	Sad	80.0
	88_12yoF_Surprise_front1	Surprised	90.0
	88_12yoF_Surprise_front2	Surprised	85.0
**94**	94_7yoF_Angry_front1	Angry	75.0
	94_7yoF_Angry_front2	Angry	70.0
	94_7yoF_Disgust_front1	Disgusted	50.0
	94_7yoF_Disgust_front2	Disgusted	15.0
	94_7yoF_Fear_front1	Afraid	25.0
	94_7yoF_Fear_front2	Afraid	60.0
	94_7yoF_front1	Neutral	90.0
	94_7yoF_front2	Neutral	60.0
	94_7yoF_HappyNoTeeth_front1	Content	100.0
	94_7yoF_HappyNoTeeth_front2	Content	95.0
	94_7yoF_HappyTeeth_front1	Happy	90.0
	94_7yoF_HappyTeeth_front2	Happy	100.0
	94_7yoF_Sad_front1	Sad	100.0
	94_7yoF_Sad_front2	Sad	95.0
	94_7yoF_Surprise_front1	Surprised	95.0
	94_7yoF_Surprise_front2	Surprised	70.0
**96**	96_12yoF_Angry_front1	Angry	70.0
	96_12yoF_Angry_front2	Angry	75.0
	96_12yoF_Disgust_front1	Disgusted	90.0
	96_12yoF_Disgust_front2	Disgusted	90.0
	96_12yoF_Fear_front1	Afraid	60.0
	96_12yoF_Fear_front2	Afraid	55.0
	96_12yoF_front	Neutral	95.0
	96_12yoF_HappyNoTeeth_front1	Content	100.0
	96_12yoF_HappyNoTeeth_front2	Content	100.0
	96_12yoF_HappyTeeth_front1	Happy	100.0
	96_12yoF_HappyTeeth_front2	Happy	95.0
	96_12yoF_Sad_front1	Sad	95.0
	96_12yoF_Sad_front2	Sad	100.0
	96_12yoF_Surprise_front1	Surprised	55.0
	96_12yoF_Surprise_front2	Surprised	65.0
**98**	98_9yoF_Angry_front1	Angry	75.0
	98_9yoF_Angry_front2	Angry	80.0
	98_9yoF_Disgust_front1	Disgusted	95.0
	98_9yoF_Disgust_front2	Disgusted	100.0

Males			
Model ID	Image ID	Intended Expression	Rating (%)
68 cont…	68_9yoM_HappyTeeth	Happy	95.0
	68_9yom_sad_front2	Sad	95.0
	68_9yom_sad	Sad	100.0
70	70_8y_surprise_front2	Surprised	80.0
	70_8y_surprise	Surprised	75.0
	70_8yoM_Angry_front2	Angry	95.0
	70_8yoM_Angry	Angry	90.0
	70_8yoM_disgust_front2	Disgusted	50.0
	70_8yoM_disgust	Disgusted	70.0
	70_8yoM_Fear_front2	Afraid	20.0
	70_8yoM_Fear	Afraid	45.0
	70_8yoM	Neutral	95.0
	70_8yoM_front2	Neutral	95.0
	70_8yoM_HappyNoTeeth_front2	Content	100.0
	70_8yoM_HappyNoTeeth	Content	85.0
	70_8yoM_HappyTeeth_front2	Happy	100.0
	70_8yoM_HappyTeeth	Happy	95.0
	70_8yom_sad_front2	Sad	95.0
	70_8yom_sad	Sad	95.0
72	72_10y_surprise_front2	Surprised	90.0
	72_10y_surprise	Surprised	90.0
	72_10yoM_Angry_front2	Angry	85.0
	72_10yoM_Angry	Angry	75.0
	72_10yoM_disgust_front2	Disgusted	95.0
	72_10yoM_disgust	Disgusted	95.0
	72_10yoM_Fear_front2	Afraid	55.0
	72_10yoM_Fear	Afraid	55.0
	72_10yoM	Neutral	95.0
	72_10yoM_front2	Neutral	90.0
	72_10yoM_HappyNoTeeth_front2	Content	100.0
	72_10yoM_HappyNoTeeth	Content	100.0
	72_10yoM_HappyTeeth_front2	Happy	100.0
	72_10yoM_HappyTeeth	Happy	100.0
	72_10yom_sad_front2	Sad	95.0
	72_10yom_sad	Sad	95.0
73	73_8y_surprise_front2	Surprised	55.0
	73_8y_surprise	Surprised	35.0
	73_8yoM_Angry_front2	Angry	90.0
	73_8yoM_Angry	Angry	80.0
	73_8yoM_disgust_front2	Disgusted	55.0
	73_8yoM_disgust	Disgusted	50.0

Females			
Model ID	Image ID	Intended Expression	Rating (%)
98 cont…	98_9yoF_Fear_front1	Afraid	45.0
	98_9yoF_Fear_front2	Afraid	35.0
	98_9yoF_front1	Neutral	85.0
	98_9yoF_front2	Neutral	85.0
	98_9yoF_HappyNoTeeth_front1	Content	80.0
	98_9yoF_HappyNoTeeth_front2	Content	90.0
	98_9yoF_HappyTeeth_front1	Happy	100.0
	98_9yoF_HappyTeeth_front2	Happy	95.0
	98_9yoF_Sad_front1	Sad	95.0
	98_9yoF_Sad_front2	Sad	65.0
	98_9yoF_Surprise_front1	Surprised	75.0
	98_9yoF_Surprise_front2	Surprised	85.0
99	99_7yoF_Angry_front1	Angry	85.0
	99_7yoF_Angry_front2	Angry	85.0
	99_7yoF_Disgust_front1	Disgusted	65.0
	99_7yoF_Disgust_front2	Disgusted	70.0
	99_7yoF_Fear_front1	Afraid	70.0
	99_7yoF_Fear_front2	Afraid	30.0
	99_7yoF_front1	Neutral	95.0
	99_7yoF_front2	Neutral	90.0
	99_7yoF_HappyNoTeeth_front1	Content	90.0
	99_7yoF_HappyNoTeeth_front2	Content	90.0
	99_7yoF_HappyTeeth_front1	Happy	100.0
	99_7yoF_HappyTeeth_front2	Happy	95.0
	99_7yoF_Sad_front1	Sad	30.0
	99_7yoF_Sad_front2	Sad	65.0
	99_7yoF_Surprise_front1	Surprised	85.0
	99_7yoF_Surprise_front2	Surprised	100.0
102	102_9yoF_Angry_front1	Angry	25.0
	102_9yoF_Disgust_front2	Disgusted	100.0
	102_9yoF_Disgust_front1	Disgusted	85.0
	102_9yoF_Fear_front1	Afraid	35.0
	102_9yoF_Fear_front2	Afraid	30.0
	102_9yoF_front1	Neutral	95.0
	102_9yoF_front2	Neutral	95.0
	102_9yoF_HappyNoTeeth_front1	Content	100.0
	102_9yoF_HappyNoTeeth_front2	Content	95.0
	102_9yoF_HappyTeeth_front1	Happy	100.0
	102_9yoF_HappyTeeth_front2	Happy	85.0
	102_9yoF_Sad_front1	Sad	55.0
	102_9yoF_Sad_front2	Sad	35.0

Males			
Model ID	Image ID	Intended Expression	Rating (%)
**73 cont…**	73_8yoM_Fear_front2	Afraid	25.0
	73_8yoM_Fear	Afraid	45.0
	73_8yoM	Neutral	95.0
	73_8yoM_front2	Neutral	95.0
	73_8yoM_HappyNoTeeth_front2	Content	95.0
	73_8yoM_HappyNoTeeth	Content	90.0
	73_8yoM_HappyTeeth_front2	Happy	100.0
	73_8yoM_HappyTeeth	Happy	100.0
	73_8yom_sad_front2	Sad	100.0
	73_8yom_sad	Sad	95.0
**75**	75_10y_surprise_front2	Surprised	95.0
	75_10y_surprise	Surprised	85.0
	75_10yoM_Angry_front2	Angry	55.0
	75_10yoM_Angry	Angry	40.0
	75_10yoM_disgust_front2	Disgusted	80.0
	75_10yoM_disgust	Disgusted	90.0
	75_10yoM_Fear_front2	Afraid	30.0
	75_10yoM_Fear	Afraid	35.0
	75_10yoM	Neutral	100.0
	75_10yoM_front2	Neutral	100.0
	75_10yoM_HappyNoTeeth_front2	Content	95.0
	75_10yoM_HappyNoTeeth	Content	100.0
	75_10yoM_HappyTeeth_front2	Happy	90.0
	75_10yoM_HappyTeeth	Happy	90.0
	75_10yom_sad_front2	Sad	95.0
	75_10yom_sad	Sad	95.0
**76**	76_10y_surprise_front2	Surprised	90.0
	76_10y_surprise	Surprised	85.0
	76_10yoM_Angry_front2	Angry	65.0
	76_10yoM_Angry	Angry	40.0
	76_10yoM_disgust_front2	Disgusted	50.0
	76_10yoM_disgust	Disgusted	65.0
	76_10yoM_Fear_front2	Afraid	75.0
	76_10yoM_Fear	Afraid	65.0
	76_10yoM_HappyNoTeeth_front2	Content	100.0
	76_10yoM_HappyNoTeeth	Content	100.0
	76_10yoM_HappyTeeth_front2	Happy	100.0
	76_10yoM_HappyTeeth	Happy	100.0
	76_10yom_sad_front2	Sad	95.0
	76_10yom_sad	Sad	95.0
	76_8yoM_front2	Neutral	100.0

Females			
Model ID	Image ID	Intended Expression	Rating (%)
**102 cont…**	102_9yoF_Surprise_front1	Surprised	85.0
	102_9yoF_Surprise_front2	Surprised	70.0
**105**	105_10yoF_Angry_front1	Angry	80.0
	105_10yoF_Angry_front2	Angry	90.0
	105_10yoF_Disgust_front1	Disgusted	45.0
	105_10yoF_Disgust_front2	Disgusted	85.0
	105_10yoF_Fear_front1	Afraid	80.0
	105_10yoF_Fear_front2	Afraid	70.0
	105_10yoF_front1	Neutral	95.0
	105_10yoF_front2	Neutral	100.0
	105_10yoF_HappyNoTeeth_front1	Content	95.0
	105_10yoF_HappyNoTeeth_front2	Content	100.0
	105_10yoF_HappyTeeth_front1	Happy	100.0
	105_10yoF_HappyTeeth_front2	Happy	95.0
	105_10yoF_Sad_front1	Sad	100.0
	105_10yoF_Sad_front2	Sad	100.0
	105_10yoF_Surprise_front1	Surprised	55.0
	105_10yoF_Surprise_front2	Surprised	45.0
**109**	109_14yoF_Angry_front1	Angry	70.0
	109_14yoF_Angry_front2	Angry	75.0
	109_14yoF_Disgust_front1	Disgusted	95.0
	109_14yoF_Disgust_front2	Disgusted	65.0
	109_14yoF_Fear_front1	Afraid	80.0
	109_14yoF_Fear_front2	Afraid	85.0
	109_14yoF_front1	Neutral	65.0
	109_14yoF_front2	Neutral	65.0
	109_14yoF_HappyNoTeeth_front1	Content	95.0
	109_14yoF_HappyNoTeeth_front2	Content	90.0
	109_14yoF_HappyTeeth_front1	Happy	100.0
	109_14yoF_HappyTeeth_front2	Happy	95.0
	109_14yoF_Sad_front1	Sad	95.0
	109_14yoF_Sad_front2	Sad	95.0
	109_14yoF_Surprise_front1	Surprised	65.0
	109_14yoF_Surprise_front2	Surprised	85.0
**110**	110_13yoF_Angry_front1	Angry	80.0
	110_13yoF_Angry_front2	Angry	95.0
	110_13yoF_Disgust_front1	Disgusted	95.0
	110_13yoF_Disgust_front2	Disgusted	85.0
	110_13yoF_Fear_front1	Afraid	40.0
	110_13yoF_Fear_front2	Afraid	45.0
	110_13yoF_front1	Neutral	95.0

Males			
**Model ID**	**Image ID**	**Intended Expression**	**Rating (%)**
**76 cont…**	76_8yoM	Neutral	95.0
**79**	79_7y_surprise_front2	Surprised	75.0
	79_7y_surprise	Surprised	70.0
	79_7yoM_Angry_front2	Angry	85.0
	79_7yoM_Angry	Angry	80.0
	79_7yoM_disgust_front2	Disgusted	80.0
	79_7yoM_disgust	Disgusted	80.0
	79_7yoM_Fear_front2	Afraid	60.0
	79_7yoM_Fear	Afraid	50.0
	79_7yoM	Neutral	100.0
	79_7yoM_front2	Neutral	100.0
	79_7yoM_HappyNoTeeth_front2	Content	100.0
	79_7yoM_HappyNoTeeth	Content	100.0
	79_7yoM_HappyTeeth_front2	Happy	95.0
	79_7yoM_HappyTeeth	Happy	100.0
	79_7yom_sad_front2	Sad	100.0
	79_7yom_sad	Sad	100.0
**80**	80_11y_surprise_front2	Surprised	90.0
	80_11y_surprise	Surprised	95.0
	80_11yoM_Angry_front2	Angry	55.0
	80_11yoM_Angry	Angry	60.0
	80_11yoM_disgust_front2	Disgusted	95.0
	80_11yoM_disgust	Disgusted	95.0
	80_11yoM_Fear_front2	Afraid	35.0
	80_11yoM_Fear	Afraid	30.0
	80_11yoM	Neutral	100.0
	80_11yoM_front2	Neutral	95.0
	80_11yoM_HappyNoTeeth_front2	Content	85.0
	80_11yoM_HappyNoTeeth	Content	100.0
	80_11yoM_HappyTeeth_front2	Happy	100.0
	80_11yoM_HappyTeeth	Happy	100.0
	80_11yom_sad_front2	Sad	80.0
	80_11yom_sad	Sad	70.0
**81**	81_8y_surprise_front2	Surprised	100.0
	81_8y_surprise	Surprised	90.0
	81_8yoM_Angry_front2	Angry	65.0
	81_8yoM_Angry	Angry	80.0
	81_8yoM_disgust_front2	Disgusted	70.0
	81_8yoM_disgust	Disgusted	75.0
	81_8yoM_Fear_front2	Afraid	60.0
	81_8yoM_Fear	Afraid	100.0

Females			
**Model ID**	**Image ID**	**Intended Expression**	**Rating (%)**
**110 cont…**	110_13yoF_front2	Neutral	80.0
	110_13yoF_HappyNoTeeth_front1	Content	95.0
	110_13yoF_HappyNoTeeth_front2	Content	90.0
	110_13yoF_HappyTeeth_front1	Happy	95.0
	110_13yoF_HappyTeeth_front2	Happy	100.0
	110_13yoF_Sad_front1	Sad	100.0
	110_13yoF_Sad_front2	Sad	95.0
	110_13yoF_Surprise_front1	Surprised	85.0
	110_13yoF_Surprise_front2	Surprised	90.0
**111**	111_13yoF_Angry_front1	Angry	80.0
	111_13yoF_Angry_front2	Angry	80.0
	111_13yoF_Disgust_front1	Disgusted	80.0
	111_13yoF_Disgust_front2	Disgusted	80.0
	111_13yoF_Disgust_front3	Disgusted	60.0
	111_13yoF_Disgust_front4	Disgusted	75.0
	111_13yoF_Fear_front1	Afraid	25.0
	111_13yoF_Fear_front2	Afraid	25.0
	111_13yoF_front1	Neutral	80.0
	111_13yoF_front2	Neutral	70.0
	111_13yoF_HappyNoTeeth_front1	Content	75.0
	111_13yoF_HappyNoTeeth_front2	Content	90.0
	111_13yoF_HappyTeeth_front1	Happy	100.0
	111_13yoF_HappyTeeth_front2	Happy	90.0
	111_13yoF_Sad_front1	Sad	100.0
	111_13yoF_Sad_front2	Sad	95.0
	111_13yoF_Surprise_front1	Surprised	35.0
	111_13yoF_Surprise_front2	Surprised	55.0
**112**	112_11yoF_angry1	Angry	95.0
	112_11yoF_angry2	Angry	75.0
	112_11yoF_content1	Content	100.0
	112_11yoF_content2	Content	100.0
	112_11yoF_disgust1	Disgusted	85.0
	112_11yoF_disgust2	Disgusted	90.0
	112_11yoF_fear	Afraid	85.0
	112_11yoF_fear2	Afraid	65.0
	112_11yoF_happy1	Happy	100.0
	112_11yoF_happy2	Happy	100.0
	112_11yoF_neutral1	Neutral	100.0
	112_11yoF_neutral2	Neutral	80.0
	112_11yoF_sad1	Sad	100.0
	112_11yoF_sad2	Sad	90.0

Males			
Model ID	Image ID	Intended Expression	Rating (%)
81 cont…	81_8yoM	Neutral	50.
	81_8yoM_front2	Neutral	90.0
	81_8yoM_HappyNoTeeth_front2	Content	60.0
	81_8yoM_HappyNoTeeth	Content	60.0
	81_8yoM_HappyTeeth_front2	Happy	95.0
	81_8yoM_HappyTeeth	Happy	95.0
	81_8yom_sad_front2	Sad	95.0
	81_8yom_sad	Sad	90.0
84	84_11y_surprise_front2	Surprised	100.0
	84_11y_surprise	Surprised	95.0
	84_11yoM_Angry_front2	Angry	25.0
	84_11yoM_Angry	Angry	25.0
	84_11yoM_disgust_front2	Disgusted	80.0
	84_11yoM_disgust	Disgusted	75.0
	84_11yoM_Fear_front2	Afraid	55.0
	84_11yoM_Fear	Afraid	45.0
	84_11yoM	Neutral	90.0
	84_11yoM_front2	Neutral	100.0
	84_11yoM_HappyNoTeeth_front2	Content	100.0
	84_11yoM_HappyNoTeeth	Content	100.0
	84_11yoM_HappyTeeth_front2	Happy	95.0
	84_11yoM_HappyTeeth	Happy	100.0
	84_11yom_sad_front2	Sad	100.0
	84_11yom_sad	Sad	95.0
86	86_7y_surprise_front2	Surprised	90.0
	86_7y_surprise	Surprised	85.0
	86_7yoM_Angry_front2	Angry	100.0
	86_7yoM_Angry	Angry	90.0
	86_7yoM_disgust_front2	Disgusted	50.0
	86_7yoM_disgust	Disgusted	65.0
	86_7yoM_Fear_front2	Afraid	20.0
	86_7yoM_Fear	Afraid	25.0
	86_7yoM	Neutral	85.0
	86_7yoM_front2	Neutral	100.0
	86_7yoM_HappyNoTeeth_front2	Content	100.0
	86_7yoM_HappyNoTeeth	Content	100.0
	86_7yoM_HappyTeeth_front2	Happy	100.0
	86_7yoM_HappyTeeth	Happy	100.0
	86_7yom_sad_front2	Sad	85.0
	86_7yom_sad	Sad	85.0
87	87_9y_surprise_front2	Surprised	55.0

Females			
Model ID	Image ID	Intended Expression	Rating (%)
112 cont…	112_11yoF_surprise1	Surprised	90.0
	112_11yoF_surprise2	Surprised	95.0
113	113_15yoF_angry1	Angry	65.0
	113_15yoF_angry2	Angry	80.0
	113_15yoF_content1	Content	90.0
	113_15yoF_content2	Content	100.0
	113_15yoF_disgust1	Disgusted	85.0
	113_15yoF_disgust2	Disgusted	95.0
	113_15yoF_fear1	Afraid	55.0
	113_15yoF_fear2	Afraid	50.0
	113_15yoF_happy1	Happy	100.0
	113_15yoF_happy2	Happy	100.0
	113_15yoF_neutral1	Neutral	90.0
	113_15yoF_neutral2	Neutral	95.0
	113_15yoF_sad1	Sad	35.0
	113_15yoF_sad2	Sad	55.0
	113_15yoF_surprise1	Surprised	95.0
	113_15yoF_surprise2	Surprised	70.0
114	114_9yoF_angry1	Angry	90.0
	114_9yoF_angry2	Angry	100.0
	114_9yoF_content1	Content	100.0
	114_9yoF_content2	Content	100.0
	114_9yoF_disgust1	Disgusted	95.0
	114_9yoF_disgust2	Disgusted	85.0
	114_9yoF_fear1	Afraid	35.0
	114_9yoF_fear2	Afraid	60.0
	114_9yoF_happy1	Happy	100.0
	114_9yoF_happy2	Happy	100.0
	114_9yoF_neutral1	Neutral	90.0
	114_9yoF_neutral2	Neutral	100.0
	114_9yoF_sad1	Sad	85.0
	114_9yoF_sad2	Sad	100.0
	114_9yoF_surprise1	Surprised	80.0
	114_9yoF_surprise2	Surprised	95.0
115	115_12yoF_angry1	Angry	95.0
	115_12yoF_angry2	Angry	100.0
	115_12yoF_content1	Content	100.0
	115_12yoF_content2	Content	100.0
	115_12yoF_disgust1	Disgusted	60.0
	115_12yoF_disgust2	Disgusted	70.0
	115_12yoF_fear1	Afraid	90.0

Males

Model ID	Image ID	Intended Expression	Rating (%)
87 cont…	87_9y_surprise	Surprised	60.0
	87_9yoM_Angry_front2	Angry	95.0
	87_9yoM_Angry	Angry	90.0
	87_9yoM_disgust_front2	Disgusted	85.0
	87_9yoM_disgust	Disgusted	75.0
	87_9yoM_Fear_front2	Afraid	70.0
	87_9yoM_Fear	Afraid	85.0
	87_9yoM	Neutral	100.0
	87_9yoM_front2	Neutral	100.0
	87_9yoM_HappyNoTeeth_front2	Content	100.0
	87_9yoM_HappyNoTeeth	Content	100.0
	87_9yoM_HappyTeeth_front2	Happy	100.0
	87_9yoM_HappyTeeth	Happy	95.0
	87_9yom_sad_front2	Sad	100.0
	87_9yom_sad	Sad	100.0
89	89_9y_surprise_front2	Surprised	100.0
	89_9y_surprise	Surprised	100.0
	89_9yoM_Angry_front2	Angry	90.0
	89_9yoM_Angry	Angry	75.0
	89_9yoM_disgust_front2	Disgusted	100.0
	89_9yoM_disgust	Disgusted	100.0
	89_9yoM_Fear_front2	Afraid	10.0
	89_9yoM_Fear	Afraid	10.0
	89_9yoM	Neutral	95.0
	89_9yoM_front2	Neutral	100.0
	89_9yoM_HappyNoTeeth_front2	Content	100.0
	89_9yoM_HappyNoTeeth	Content	100.0
	89_9yoM_HappyTeeth_front2	Happy	95.0
	89_9yoM_HappyTeeth	Happy	95.0
	89_9yom_sad_front2	Sad	100.0
	89_9yom_sad	Sad	85.0
92	92_8y_surprise_front2	Surprised	66.7
	92_8y_surprise	Surprised	71.4
	92_8yoM_Angry_front2	Angry	90.5
	92_8yoM_Angry	Angry	81.0
	92_8yoM_disgust_front2	Disgusted	33.3
	92_8yoM_disgust	Disgusted	28.6
	92_8yoM_Fear_front2	Afraid	57.1
	92_8yoM_Fear	Afraid	52.4
	92_8yoM	Neutral	100.0
	92_8yoM_front2	Neutral	95.2

Females

Model ID	Image ID	Intended Expression	Rating (%)
115 cont…	115_12yoF_fear2	Afraid	90.0
	115_12yoF_happy1	Happy	100.0
	115_12yoF_happy2	Happy	100.0
	115_12yoF_neutral1	Neutral	95.0
	115_12yoF_neutral2	Neutral	85.0
	115_12yoF_sad1	Sad	100.0
	115_12yoF_sad2	Sad	95.0
	115_12yoF_surprise1	Surprised	80.0
	115_12yoF_surprise2	Surprised	75.0
117	117_9yoF_angry1	Angry	75.0
	117_9yoF_angry2	Angry	10.0
	117_9yoF_content1	Content	100.0
	117_9yoF_content2	Content	95.0
	117_9yoF_disgust1	Disgusted	75.0
	117_9yoF_disgust2	Disgusted	80.0
	117_9yoF_fear1	Afraid	50.0
	117_9yoF_fear2	Afraid	70.0
	117_9yoF_happy1	Happy	100.0
	117_9yoF_happy2	Happy	100.0
	117_9yoF_neutral1	Neutral	85.0
	117_9yoF_neutral2	Neutral	95.0
	117_9yoF_sad1	Sad	100.0
	117_9yoF_sad2	Sad	100.0
	117_9yoF_surprise1	Surprised	75.0
	117_9yoF_surprise2	Surprised	85.0
119	119_14yoF_angry1	Angry	95.0
	119_14yoF_angry2	Angry	95.0
	119_14yoF_content1	Content	100.0
	119_14yoF_content2	Content	100.0
	119_14yoF_disgust1	Disgusted	70.0
	119_14yoF_disgust2	Disgusted	70.0
	119_14yoF_fear1	Afraid	65.0
	119_14yoF_fear2	Afraid	70.0
	119_14yoF_happy1	Happy	100.0
	119_14yoF_happy2	Happy	95.0
	119_14yoF_neutral1	Neutral	80.0
	119_14yoF_neutral2	Neutral	85.0
	119_14yoF_sad1	Sad	100.0
	119_14yoF_sad2	Sad	95.0
	119_14yoF_surprise1	Surprised	90.0
	119_14yoF_surprise2	Surprised	95.0

Males			
**Model ID**	**Image ID**	**Intended Expression**	**Rating (%)**
**92 cont…**	92_8yoM_HappyNoTeeth_front2	Content	100.0
	92_8yoM_HappyNoTeeth	Content	100.0
	92_8yoM_HappyTeeth_front2	Happy	100.0
	92_8yoM_HappyTeeth	Happy	100.0
	92_8yom_sad_front2	Sad	95.2
	92_8yom_sad	Sad	95.2
**103**	103_12y_surprise_front2	Surprised	90.5
	103_12y_surprise	Surprised	100.0
	103_12yoM_Angry_front2	Angry	23.8
	103_12yoM_Angry	Angry	23.8
	103_12yoM_disgust_front2	Disgusted	90.5
	103_12yoM_disgust	Disgusted	95.2
	103_12yoM_Fear_front2	Afraid	52.4
	103_12yoM_Fear	Afraid	71.4
	103_12yoM	Neutral	95.2
	103_12yoM_front2	Neutral	90.5
	103_12yoM_HappyNoTeeth_front2	Content	85.7
	103_12yoM_HappyNoTeeth	Content	100.0
	103_12yoM_HappyTeeth_front2	Happy	100.0
	103_12yoM_HappyTeeth	Happy	100.0
	103_12yom_sad_front2	Sad	71.4
	103_12yom_sad	Sad	85.7
**104**	104_12y_surprise_front2	Surprised	76.2
	104_12y_surprise	Surprised	76.2
	104_12yoM_Angry_front2	Angry	90.5
	104_12yoM_Angry	Angry	95.2
	104_12yoM_disgust_front2	Disgusted	95.2
	104_12yoM_disgust	Disgusted	95.2
	104_12yoM_Fear_front2	Afraid	66.7
	104_12yoM_Fear	Afraid	61.9
	104_12yoM	Neutral	85.7
	104_12yoM_front2	Neutral	100.0
	104_12yoM_HappyNoTeeth_front2	Content	100.0
	104_12yoM_HappyNoTeeth	Content	95.2
	104_12yoM_HappyTeeth_front2	Happy	100.0
	104_12yoM_HappyTeeth	Happy	100.0
	104_12yom_sad_front2	Sad	90.5
	104_12yom_sad	Sad	90.5
**107**	107_14y_surprise_front2	Surprised	85.7
	107_14y_surprise	Surprised	95.2
	107_14yoM_Angry_front2	Angry	52.4

Females			
**Model ID**	**Image ID**	**Intended Expression**	**Rating (%)**
**120**	120_9yoF_angry1	Angry	100.0
	120_9yoF_angry2	Angry	100.0
	120_9yoF_content1	Content	100.0
	120_9yoF_content2	Content	75.0
	120_9yoF_disgust1	Disgusted	90.0
	120_9yoF_disgust2	Disgusted	90.0
	120_9yoF_fear1	Afraid	70.0
	120_9yoF_fear2	Afraid	60.0
	120_9yoF_happy1	Happy	100.0
	120_9yoF_happy2	Happy	100.0
	120_9yoF_neutral1	Neutral	90.0
	120_9yoF_neutral2	Neutral	100.0
	120_9yoF_sad1	Sad	45.0
	120_9yoF_sad2	Sad	55.0
	120_9yoF_surprise1	Surprised	80.0
	120_9yoF_surprise2	Surprised	95.0
**121**	121_7yoF_angry1	Angry	10.0
	121_7yoF_angry2	Angry	30.0
	121_7yoF_content1	Content	75.0
	121_7yoF_content2	Content	80.0
	121_7yoF_disgust1	Disgusted	95.0
	121_7yoF_disgust2	Disgusted	95.0
	121_7yoF_fear1	Afraid	70.0
	121_7yoF_fear2	Afraid	70.0
	121_7yoF_happy1	Happy	100.0
	121_7yoF_happy2	Happy	100.0
	121_7yoF_neutral1	Neutral	100.0
	121_7yoF_neutral2	Neutral	100.0
	121_7yoF_sad1	Sad	30.0
	121_7yoF_sad2	Sad	25.0
	121_7yoF_surprise1	Surprised	90.0
	121_7yoF_surprise2	Surprised	90.0
**122**	122_16yoF_angry1	Angry	100.0
	122_16yoF_angry2	Angry	90.0
	122_16yoF_content1	Content	90.0
	122_16yoF_content2	Content	100.0
	122_16yoF_disgust1	Disgusted	85.0
	122_16yoF_disgust2	Disgusted	90.0
	122_16yoF_fear1	Afraid	65.0
	122_16yoF_fear2	Afraid	65.0
	122_16yoF_happy1	Happy	95.0

Males			
Model ID	Image ID	Intended Expression	Rating (%)
107 cont…	107_14yoM_Angry	Angry	19.0
	107_14yoM_disgust_front2	Disgusted	81.0
	107_14yoM_disgust	Disgusted	81.0
	107_14yoM_Fear_front2	Afraid	81.0
	107_14yoM_Fear	Afraid	71.4
	107_14yoM	Neutral	100.0
	107_14yoM_front2	Neutral	100.0
	107_14yoM_HappyNoTeeth_front2	Content	71.4
	107_14yoM_HappyNoTeeth	Content	71.4
	107_14yoM_HappyTeeth_front2	Happy	100.0
	107_14yoM_HappyTeeth	Happy	100.0
	107_14yom_sad_front2	Sad	90.5
	107_14yom_sad	Sad	85.7
108	108_10y_surprise_front2	Surprised	76.2
	108_10y_surprise	Surprised	81.0
	108_10yoM_Angry_front2	Angry	81.0
	108_10yoM_Angry	Angry	81.0
	108_10yoM_disgust_front2	Disgusted	81.0
	108_10yoM_disgust	Disgusted	90.5
	108_10yoM_Fear_front2	Afraid	57.1
	108_10yoM_Fear	Afraid	61.9
	108_10yoM	Neutral	95.2
	108_10yoM_front2	Neutral	95.2
	108_10yoM_HappyNoTeeth_front2	Content	61.9
	108_10yoM_HappyNoTeeth	Content	95.2
	108_10yoM_HappyTeeth_front2	Happy	100.0
	108_10yoM_HappyTeeth	Happy	100.0
	108_10yom_sad_front2	Sad	90.5
	108_10yom_sad	Sad	81.0

Females			
Model ID	Image ID	Intended Expression	Rating (%)
122 cont…	122_16yoF_happy2	Happy	100.0
	122_16yoF_neutral1	Neutral	100.0
	122_16yoF_neutral2	Neutral	70.0
	122_16yoF_sad1	Sad	80.0
	122_16yoF_sad2	Sad	80.0
	122_16yoF_surprise1	Surprised	100.0
	122_16yoF_surprise2	Surprised	95.0
123	123_16yoF_angry1	Angry	95.0
	123_16yoF_angry2	Angry	85.0
	123_16yoF_content1	Content	100.0
	123_16yoF_content2	Content	100.0
	123_16yoF_disgust1	Disgusted	85.0
	123_16yoF_disgust2	Disgusted	60.0
	123_16yoF_fear1	Afraid	45.0
	123_16yoF_fear2	Afraid	65.0
	123_16yoF_happy1	Happy	100.0
	123_16yoF_happy2	Happy	100.0
	123_16yoF_neutral1	Neutral	100.0
	123_16yoF_neutral2	Neutral	100.0
	123_16yoF_sad1	Sad	95.0
	123_16yoF_sad2	Sad	90.0
	123_16yoF_surprise1	Surprised	100.0
	123_16yoF_surprise2	Surprised	95.0
